# Revolutionizing bone healing: the role of 3D models

**DOI:** 10.1186/s13619-025-00225-1

**Published:** 2025-03-21

**Authors:** Raffaella De Pace, Maria Rosa Iaquinta, Assia Benkhalqui, Antonio D’Agostino, Lorenzo Trevisiol, Riccardo Nocini, Chiara Mazziotta, John Charles Rotondo, Ilaria Bononi, Mauro Tognon, Fernanda Martini, Elisa Mazzoni

**Affiliations:** 1 Department of Chemical, Pharmaceutical and Agricultural Sciences, University of Ferrara, Ferrara, 44121 Italy; 2 Department of Medical Sciences, University of Ferrara, Ferrara, Italy; 3 University Center for Studies On Gender Medicine, University of Ferrara, Ferrara, Italy; 4 Department of Surgery, University of Verona, Verona, Italy; 5 Centre for Medical Sciences (CISMed), University of Trento, Trento, Italy; 6 Unit of Maxillofacial Surgery, Santa Chiara Regional Hospital, APSS, Trento, Italy; 7 Centralized Laboratory of Pre-Clinical Research, University of Ferrara, Ferrara, Italy; 8 Laboratory for Technologies of Advanced Therapies (LTTA), University of Ferrara, Ferrara, Italy

**Keywords:** Bone regeneration, Cell-ECM interaction, Stem cell, Biomaterial, 3D in vitro model, Microfluidic

## Abstract

The increasing incidence of bone diseases has driven research towards Bone Tissue Engineering (BTE), an innovative discipline that uses biomaterials to develop three-dimensional (3D) scaffolds capable of mimicking the natural environment of bone tissue. Traditional approaches relying on two-dimensional (2D) models have exhibited significant limitations in simulating cellular interactions and the complexity of the bone microenvironment. In response to these challenges, 3D models such as organoids and cellular spheroids have emerged as effective tools for studying bone regeneration. Adult mesenchymal stem cells have proven crucial in this context, as they can differentiate into osteoblasts and contribute to bone tissue repair. Furthermore, the integration of composite biomaterials has shown substantial potential in enhancing bone healing. Advanced technologies like microfluidics offer additional opportunities to create controlled environments for cell culture, facilitating more detailed studies on bone regeneration. These advancements represent a fundamental step forward in the treatment of bone pathologies and the promotion of skeletal health. In this review, we report on the evolution of in vitro culture models applied to the study of bone healing/regrowth, starting from 2 to 3D cultures and microfluids. The different methodologies of in vitro model generation, cells and biomaterials are presented and discussed.

## Background

Bone diseases, both acute and chronic, are rising alongside life expectancy, with over 20 million people annually suffering from bone tissue loss (Iaquinta et al. 2019). The damaged skeletal organ is usually returned to its pre-injury state by bone fracture repair; nevertheless, 10% of fractures do not heal properly. Indeed, in some cases, osteosarcoma, osteoporosis, osteomalacia, osteomyelitis, avascular necrosis, and atrophic non-union could impair bone regeneration process (Iaquinta et al. 2019). Thus, reconstruction of bone abnormalities is therefore still a significant clinical problem. New bone tissue engineering (BTE) approaches might increase the ability to regenerate bone defects and reduce morbidity while guaranteeing damage healing (Luby et al. 2019).

**Fig. 1:**
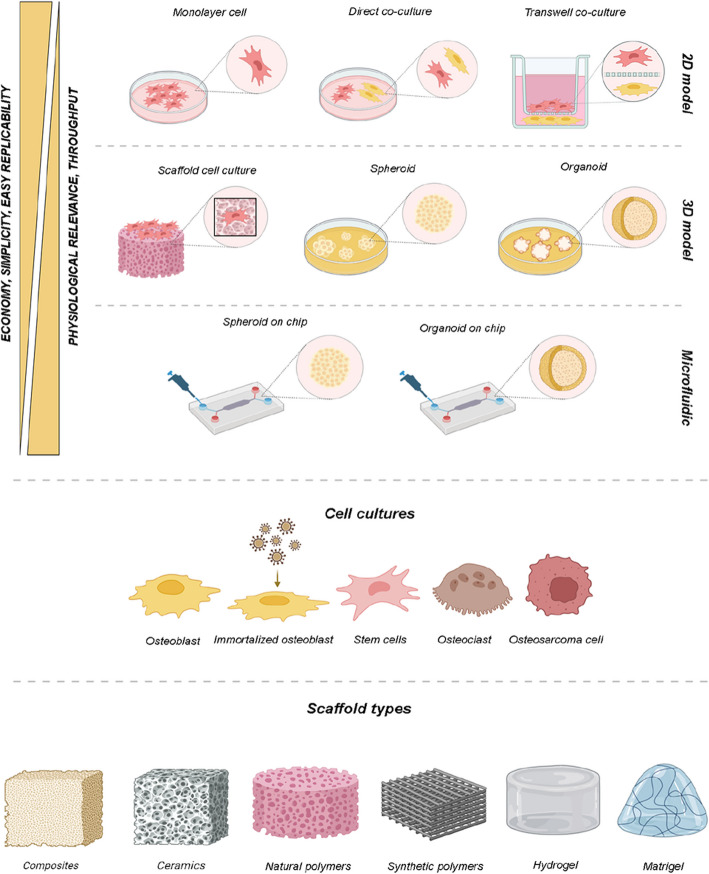
Bone regeneration research: from 2 to 3D in vitro models. Historically, 2D in vitro models were used to study bone physiology and pathology. Monolayer cultures involve cells growing in a single layer on a flat surface, while co-culture models facilitate communication between different cell types. Co-cultures can be direct, allowing physical interaction, or indirect, using transwell membranes to separate cells while permitting medium exchange. 3D models better mimic the morphology and metabolic processes of native bone tissue, promoting osteogenic differentiation. Growing cells on biomaterials creates a complex 3D microenvironment with cell–matrix interactions and mechanical properties comparable to bone. Advanced 3D models include bone organoids cultured in scaffolds that support their growth and self-organization, and spheroids which are aggregates of cells forming a sphere. Microfluidic-based spheroid/organoid-on-chips incorporate miniaturized cell-culturing environments with microchannels and compartments that replicate the natural cell environment, with potential for studying bone regeneration and orthopedic diseases. 2D culture is cheap, simple, and standardized, but lacks the complexity of native tissues. 3D cultures and microfluidics offer higher physiological relevance and throughput, but are more complex, costly, and technically challenging. Bone research models use osteoblastic and osteoclastic mammalian cell cultures from humans and animals, malignant osteosarcoma cell lines, virus immortalized osteoblasts, and human stem cells, particularly induced pluripotent stem cells and mesenchymal stem cells, are promising sources for osteoblast progenitors. Biomaterials like polymers, ceramics, and composites are ideal for bone grafting, while Matrigel and hydrogels are great for encapsulating spheroids and organoids due to their natural ECM-like characteristics

A novel strategy for bone regeneration is the use of biomaterials. With scaffolds, it is possible to develop a complex three-dimensional (3D) microenvironment analogous to the mechanical and porosity properties of bone, as well as the interactions between cells and matrix (Iaquinta et al. 2021). The gold standard material is hydroxyapatite, because it naturally forms the bone. However, a numerous types of materials, including composites, metals, ceramics, and polymers, have been created and tested (Yuste et al. 2021b). In vitro testing is necessary for the clinical evaluation of scaffolding for BTE (Fig. 1). Historically, two-dimensional (2D) in vitro models have been used to analyze physiologic and pathologic processes associated with bone and to assess the effectiveness of drugs (Boscaro, Sikorski 2024). Preclinical bone research mainly employs 2D primary cultures of osteoblast, osteoblast precursors, mesenchymal stem cells (MSCs) or induced pluripotent stem cells (iPSCs), immortalized, and malignant cell lines in order to recreate bone microenvironment. Immortalized cells cannot represent the entire phenotypic spectrum of normal osteoblasts and prolonged passages lead to phenotypic heterogeneity, while the main limit of cancer cells is that they can manifest aberrations typically tumor and genetic drift caused by heteroploidia (Yuste et al. 2021b).

At present, the best in vitro model for studying bone regeneration is represented by human stem cells. Studies show that adult stem cells (ASCs), including induced pluripotent stem cells (iPSCs) and human mesenchymal stem cells (hMSCs), are excellent osteoblast progenitors, aiding wound healing and bone repair (Luby et al. 2019). iPSCs can be generated by reprogramming human fibroblasts and inducing osteoblastic differentiation with specific nuclear transcription factors. However, generating iPSCs can be complex, costly and time-consuming, with low reprogramming efficiency and potential gene expression alterations (Ansari et al. 2021). The ability of hMSCs to differentiate into osteoprogenitors is regulated by Runt-related transcription factor 2 (Runx2) and Osterix (Sp7), which control gene expression and determine the osteoblast phenotype (Stamnitz, Klimczak 2021). The most widely used hMSCs in clinical practice are isolated from adipose tissues (hADSCs) and bone marrow (hBMSCs). These stem cells are favored in tissue engineering due to their high expansion rate, immune modulation property, and ability to repair damaged tissue (Abu-El-Rub et al. 2024). Monolayer cell cultures are inexpensive, simple to manage, and facilitate easy replication and standardization of research. However, cells grown in 2D models exhibit simplistic interactions that fail to recreate the natural bone niche microenvironment, leading to inaccurate results.

Co-culture models offer advantages over monolayer cultures by enabling intercellular signal transmission through junctions, exosomes, and autocrine/paracrine activities between various cell types, which can provide cells with new functionalities (Borciani et al. 2020). Compared to monolayer cultures, 3D cells cultures have demonstrated a distinct set of advantages and innovations, including higher cell survival, comparable in vivo morphology, and better cell–cell and cell-extracellular matrix (ECM) interactions (Borciani et al. 2020).

Cell spheroids in vitro models are three-dimensional structures that mimic the physiological tissue microenvironment and different techniques can be used to manufacture them. Spheroids can be a useful model to investigate bone and associated biological processes because of their significant ECM synthesis and cell-ECM interactions (Boscaro, Sikorski 2024).

3D bone organoids in vitro culture systems can strongly simulate the precise position and spatial morphology of cells and matrix, demonstrating cell–matrix interaction effects (Li et al. 2023). Materials made of bone organs are easily obtained and generally available. Through organoids, all aspects of the bone regeneration process can be replicated by cell expanding or differentiating while maintaining a stable phenotype. Furthermore, bone organoids can promote osteogenesis and stimulate bone regeneration by using appropriate cytokines or bioactive substances (Li et al. 2023).

Microfluidics, a new cell culture technology enabled by micro and nanoscale manufacturing advancements, allows the creation of intricate microscale structures that mimic in vivo environments. Microfluidic chips, or lab-on-a-chip devices, feature well-defined, controllable microenvironments, ensuring ideal, repeatable, and controlled cell culture growth (Li et al. 2012).

## Differences and relationships among bone cellular models

Biomaterials form the base of all 3D models. These constructs act as physical support for cell growth, providing a three-dimensional microenvironment that mimics the extracellular matrix (ECM). They are used in both simple models like bone spheroids and more complex ones like organoids and on-chip systems. For example, hydroxyapatite and collagen are frequently used to enhance cell differentiation into osteoblasts in spheroid and organoid models. Unlike other models, biomaterials fail to fully simulate the biological dynamics and physiological responses, making them less suitable for research on complex cell interactions and disease models.

Transwell and Co-culture Systems provide an environment in which different cell types can interact, simulating the physical and chemical signals typical of the in vivo bone microenvironment. They are often combined with biomaterials to improve cell adhesion and facilitate cell-to-cell interactions. They support models like spheroids and organoids to increase microenvironment complexity. Unlike other models, these systems are limited to simulating cell interactions and do not offer full three-dimensionality or the flow dynamics that more advanced systems can provide.

Bone Spheroids are simple three-dimensional structures that mimic bone tissue due to their ability to replicate cell–matrix interactions and ECM synthesis. Biomaterials improve the stability and mechanical properties of spheroids, making them more similar to real bone tissue. Moreover, biomaterials are used in combination with co-culture systems to study the interaction between different cell types. Compared to organoids, spheroids are less complex: they cannot replicate specific functions like vessel formation or bone metabolism. However, they are easier to produce and offer a versatile platform for less complex experiments.

Bone Organoids represent a higher level of complexity, mimicking not only bone structure but also bone functions such as regeneration and metabolism. The organoids require more advanced biomaterials to ensure a stable and appropriate microenvironment. They can be co-cultured with other cell types to simulate complex interactions, such as those between osteoblasts and osteoclasts. Compared to spheroids, organoids are more complex and difficult to create, but they provide a much more faithful simulation of in vivo conditions. Unlike on-chip models, they do not integrate dynamic flows.

Spheroid-on-chip combines spheroids with microfluidic technologies, adding the ability to simulate dynamic flows and mechanical stresses typical of the in vivo bone environment. It provides an advanced platform for studying how nutrients, chemical signals, and mechanical stress influence bone formation and regeneration.

Organoid-on-chip are the most advanced model, combining organoids and microfluidics, simulating both the three-dimensional structure and dynamic flows of the bone environment. It allows replication of complex functions such as bone formation, metabolism, and interaction with other tissues (e.g., innervation or vascularization). It requires sophisticated biomaterials to maintain the stability and integrity of the model. Compared to other models, it represents the highest level of simulation of in vivo bone conditions, but it is more expensive and technically more complex to create.

**Fig. 2:**
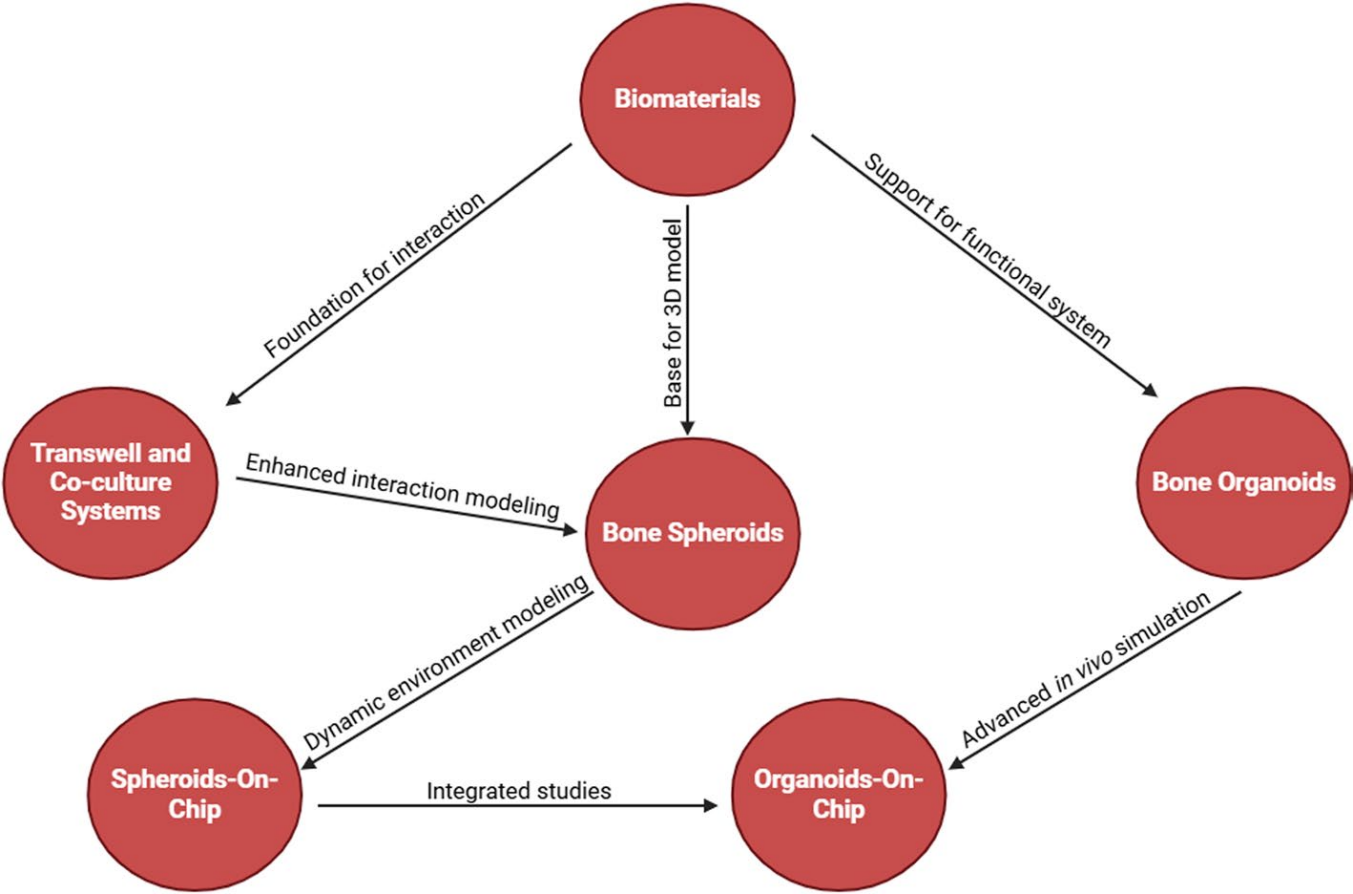
Hierarchical diagram of the models for studying bone tissue. The diagram uses a pyramidal structure to show the logical progression and increasing complexity among the different approaches for studying bone tissue. The Biomaterials form the base of the pyramid. They provide essential physical and chemical support for cell growth, differentiation, and the creation of three-dimensional microenvironments. Transwell and Co-culture Systems represent an intermediate step in complexity. They facilitate cell-to-cell interactions and mutual influence through soluble and physical signals, creating a more realistic microenvironment. Bone Spheroids offer an advanced three-dimensional model, enabling the study of cell-extracellular matrix interactions and simulating the bone microenvironment in vitro. Bone Organoids are the higher level in terms of biological simulation. They replicate more complex structures and functions, such as bone formation and regeneration. Spheroids-on-chip integrate spheroids into a dynamic system that simulates in vivo conditions, such as nutrient flow and mechanical signals. Organoids-on-chip represent the most advanced level. It combines organoids with microfluidic technologies to recreate highly specific physiological microenvironments, providing a platform for complex and personalized studies. The transition from 2 to 3D models and ultimately to chip-based systems is driven by the need to better replicate the biological environment of bone, enabling more realistic models for studying bone regeneration and developing innovative therapies. Each step in the progressive evolution of techniques used to study bone—from bidimensional models to advanced technologies like organoids, spheroids, and chip systems—is based on identifying specific limitations of the previous model and addressing them through innovative approaches

The differences among the models are primarily due to the level of complexity and their ability to simulate in vivo conditions: Biomaterials and Transwell/Co-culture systems are fundamental for building the base of three-dimensional models. Spheroids and organoids represent more complex three-dimensional models, with the former focused on cell-ECM interactions and the latter on the representation of specific tissue functions. Spheroid-on-chip and Organoid-on-chip integrate dynamics and fluid dynamics to come even closer to physiological conditions. These models are not independent, but often integrate with each other to study the bone microenvironment and regenerative processes more comprehensively (Fig. 2).

## Bone structure and organization

Bone is a well-organized, dynamic, and metabolically active tissue composed of an organic matrix comprising lipids, collagen, non-structural proteins, glycosaminoglycans, and the hydroxyapatite mineral phase (Yuste et al. 2021a).

**Fig. 3:**
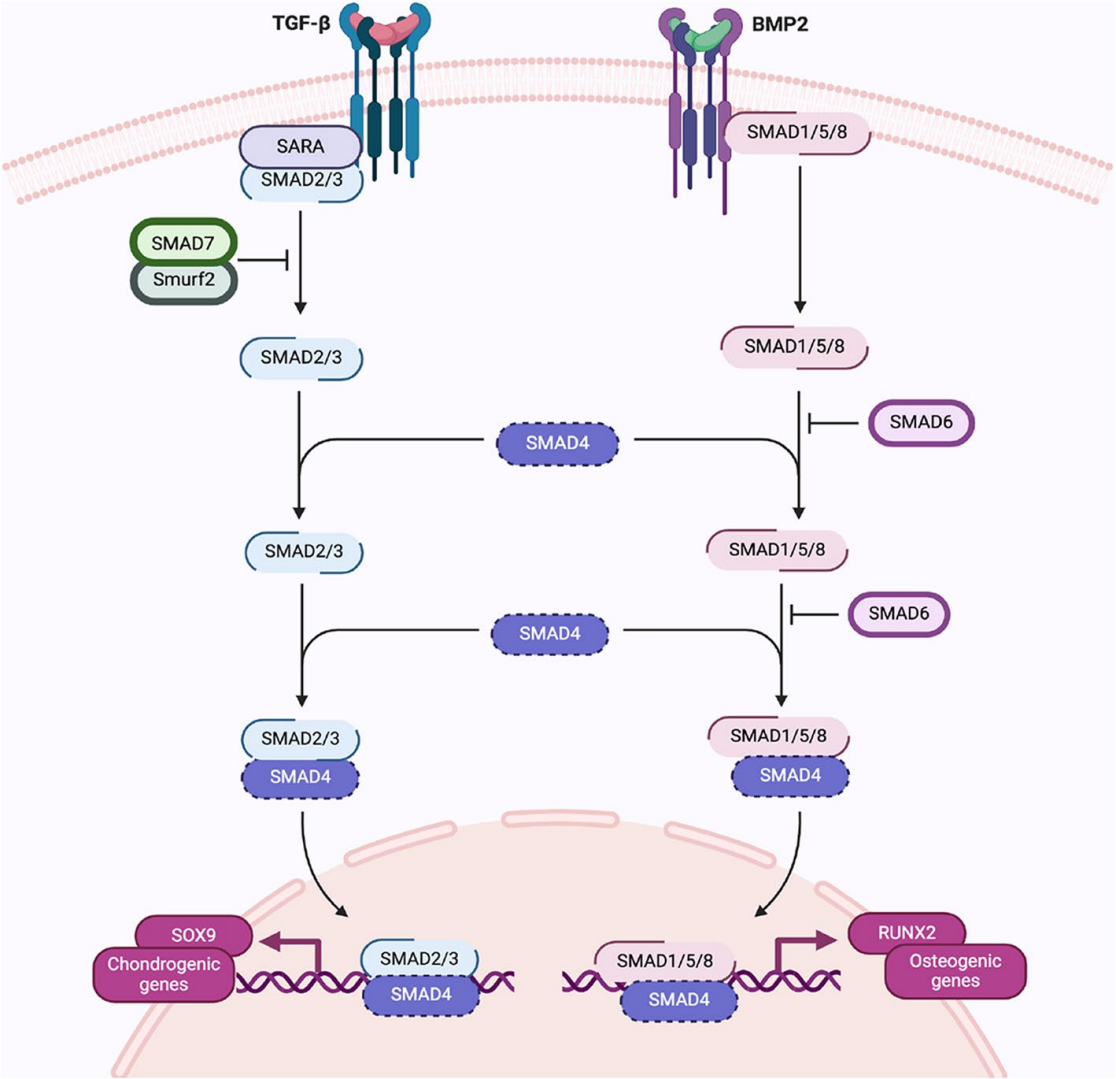
Signaling pathways of TGF-β and BMP. The members of the TGFβ and BMP families are involved in bone development, extracellular matrix and cartilage maturation. TGF-β and BMP bind to the extracellular domains of specific receptors and require SMAD proteins for signal transduction within cells. TGF-β and BMP act through heterodimer receptors made up of kinase proteins type I and II. After ligand binding to the receptor, the receptor forms homodimeric complexes, which can auto phosphorylate serine/threonine residues. This triggers a cascade of events involving the phosphorylation of the SMAD protein. The TGF-β pathway requires SMAD2 and SMAD3 which react with SMAD4 to create a heterocomplex. This complex enters the nucleus and controls transcription by binding to target gene promoters including SOX9 and other genes involved in the chondrogenesis mechanism. SMAD7 is an adaptor protein that recruits ubiquitin ligases, called Smurfs and binds them to the TGF-β receptor complex to promote its degradation through proteasomal and lysosomal pathways. Therefore, Smad7 plays a crucial role in a negative feedback cycle to control TGF-β activity. The BMP2 signal depends on SMAD1, 5 and 8. They bind SMAD4 to move into the core, where they induce the expression of RUNX2 and other genes leading to differentiation of osteoblasts and osteocytes

Bone cells are divided into (i) osteoblasts, which are mononuclear cells responsible for the synthesis, deposition, and formation of bone matrix proteins; (ii) osteocytes, which are mature osteoblasts trapped in small cavities of bone matrix called lacunae; (iii) bone lining cells, which are flat, elongated osteoblasts that cover bone surfaces and lack synthetic functions; (iv) pre-osteoblasts, which are osteoblast mesenchymal cell precursors and (v) osteoclasts, which are large multinucleated cells responsible for bone resorption, their precursors are mononuclear hematopoietic cells (Yuste et al. 2021b). The signaling pathways of beta-transforming growth factor (TGF-β) and bone morphogenic proteins (BMPs) control both osteoblast and osteoclast functions and play a critical role in bone remodeling process. The TGF-β/BMP pathway is an important signaling pathway that regulates the differentiation of hMSCs. This pathway has widely recognized roles in bone formation during mammalian development (Zou et al. 2021) (Fig. 3).

**Fig. 4:**
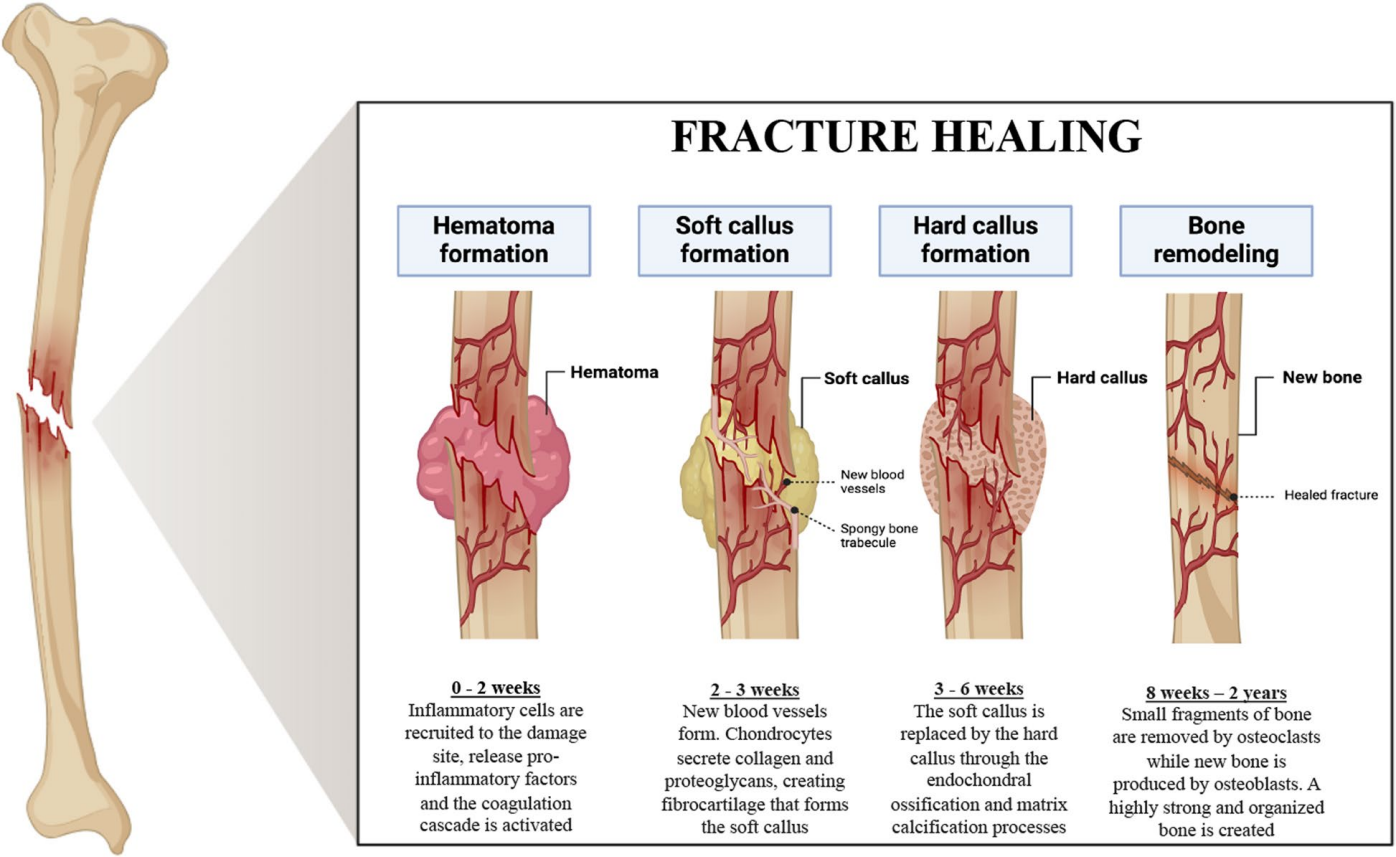
Stages of bone fracture healing. After a bone fracture, an inflammatory response occurs that lasts for two weeks. This phase starts an intricate network of proinflammatory signals and growth factors. Polymorphonucleate (PMN) cells and macrophages are recruited to endocyte microdebris and micro-organisms derived from the fracture. The damage to the blood vessels results in edema. After 2–3 weeks from the fracture, endocondral bone formation occurs. During this process, the MSCs are recruited in the injured site and begin to differentiate into chondroblasts (condrogenesis), which proliferate into chondrocytes, resulting in soft calluses. Chondrocytes synthesize and secrete the cartilage matrix, containing type II collagen and proteoglycans. Between the third and sixth week, the cartilage undergoes hypertrophy and mineralization in a spatially organized way. New MSCs are recruited which differentiate into osteoblasts, leading to the formation of interwoven bone (hard callus). Mineralized bone formation is induced by the signaling of factors such as BMP, TGF-β 2 and -β 3 in the cartilaginous callus. The last phase of bone remodeling begins 8 weeks after fracture and can last up to 2 years. Communication between osteoclasts and osteoblasts, during this phase, mediates the replacement of the braided bone with lamellar bone through two key activities: removal of the bone (resorption) by the resulting osteoclasts of the hematopoietic line and formation of the bone matrix by the mesenchymal line osteoblasts

Bone tissue self-regenerates not only after damage, but also during normal cell turnover to maintain structural integrity and functionality (Majidinia et al. 2018). Bone fracture repair is literally considered as a regenerative process that leads to the formation of new uninjured bone. The healing process consists of four overlapping phases: inflammation, fibrocartilaginous callus formation, mineralized bone callus formation, and remodeling (Boyce 2013) (Fig. 4). Initially, a hematoma forms, releasing pro-inflammatory cytokines and recruiting macrophages and polymorphonuclear leukocytes (PMNs). Then, the fibrocartilaginous callus undergoes mineralization, becoming bony callus, involving chondrocytes, osteoblasts, and osteoclasts. Remodeling is the final phase, balancing the activity of osteocytes, osteoblasts, and osteoclasts (Sheen et al. 2024).

## Employed cells for 2D in vitro model

Preclinical models in bone research mostly utilize 2D mammalian cell cultures. Monolayer cell cultures are inexpensive, simple to maintain, and have simply repeatable and standardized analysis. Osteoblasts are the primary cell type found in bones and most research uses these cells. Nowadays, stem cells, malignant cell lines and immortalized cells are also used to create in vitro study models (Yuste et al. 2021b).

The isolation site, gender, and age of the donor have an impact on primary bone cells activity. Osteoblast proliferation is reduced in older adults, postmenopausal women, and in specific bones sites like the femoral head (Aibar-Almazán et al. 2022).

Osteoblast cell lines can be isolated from tumoral bone tissue, e.g. osteosarcoma. Osteogenic potential is preserved by these cells, but the main disadvantages are heteroploidy-induced genetic drift and characteristic tumor cell aberrations. SAOS-2, OHS-4, HOS-TE-85, MG-63, KPD-XM, TPXM, and CAL72 are the human osteosarcoma cell lines (Yuste et al. 2021b). Another cell model is based on the genetically modified SAOS-2 human osteosarcoma cell line so that they constitutively express, as reported, the advanced green fluorescent protein (eGFP). The engineered cell line was called SAOS-eGFP (Globig et al. 2020). Osteoblasts are immortalized through the insertion of the large SV40 T antigen cDNA of recombinant retrovirus (Yuste et al. 2021b). High cell production is a benefit of immortalized lines but extended passages result in increasing phenotypic heterogeneity and an inability to mimic the phenotype of normal osteoblasts (Kartsogiannis, Ng 2004).

The best in vitro model for studying bone regenerative therapy is human stem cells. More recent studies have demonstrated that adult stem cells (ASCs) have a high level of differentiation and contribute to wound healing, soft tissue regeneration, and bone repair (Luby et al. 2019). Many types of adult stem cells, including human embryonic stem cells (hESCs), induced pluripotent stem cells (iPSCs) and human mesenchymal stem cells (hMSCs) have been identified as a source of osteoblast progenitors. The disintegration of the human embryo is necessary for the isolation of hESCs, for this reason their use is highly debated (Iaquinta et al. 2019).

It is possible to generate iPSCs by reprogramming human fibroblasts by transferring a mixture of nuclear transcriptional factors including octamer binding transcription factor 4 (Oct 4), sex-determining region Y-box 2 (Sox2), Kruppel-like factor 4 (Klf4) and c-Myc. By growing iPSCs and adding osteoblast-specific transcription factors, such as Runx2 and Sp7, the differentiation can be performed (Ansari et al. 2021). Several studies have demonstrated the ability of iPSCs to differentiate into osteoblasts, suggesting that iPSCs could be considered as a cellular model for in vitro bone regeneration (Kang et al. 2016). However, approaches to generating iPSCs could be complex, costly and time-consuming with low reprogramming efficiency and possible alternations of profiles and pathways of gene expression (Ansari et al. 2021).

For their paracrine in vivo activities, MSCs attract a lot of interest. MSCs release a variety of cytokines and chemokines, including transforming growth factor beta1 (TGFβ1), insulin growth factor 1 (IGF1), and fibroblast growth factor 2 (FGF2). The resident cells can get advantages from these paracrine effects, which include immunomodulation, anti-apoptotic, and anti-oxidative effects (Molnar et al. 2022). The ability of MSCs to differentiate into osteoprogenitors is one of their most significant functions for bone regeneration. Osteogenic differentiation includes the activation of Runx2 and Sp7 transcription factors, which control gene expression and determine the osteoblasts phenotype (Stamnitz e Klimczak 2021). Alkaline phosphatase (ALP) is the early osteogenic marker-protein expressed in committed osteoprogenitors; osteocalcin (OCN), osteonectin (SPARC) and osteopontin (OPN) are expressed by mature osteoblasts (Millan et al. 2018).

Recent research demonstrated that MSCs can be isolated from a wide spectrum of adult and fetal tissues, including amniotic fluid (AF-MSCs), dental pulp tissues (DPSCs), placental-derived MSCs (PD-MSCs), bone marrow (BMSCs) and adipose tissues (ADSCs) (Liao 2014). The most commonly used MSCs in clinical practice are isolated from adipose tissues and bone marrow. Many previous data support the use of these cells in tissue engineering due to their elevated rate of expansion, ability to modulate the immune system and to achieve the damaged tissue (Abu-El-Rub et al. 2024).

Higher yields of ADSCs can be obtained from the subcutaneous area easily and painless and these cells are the best candidate for allogeneic transplantation. ADSCs can be induced into osteogenic lineage by bioactive molecules, and their secretions, especially exosomes or EVs (extracellular vesicles carrying proteins, RNA, DNA, and lipid molecules), are related to fracture healing (Fröbel et al. 2021).

BMSCs have numerous advantageous characteristics for regenerative therapy, including immunomodulatory, multipotential, and anti-inflammatory qualities (Nguyen et al. 2013). These stem cells can also promote angiogenesis and assist hematopoiesis. Furthermore, the external microenvironment is impacted by the paracrine substances released by the BMSC, which is very important for organ repair (Arthur, Gronthos 2020). It has been demonstrated that BMSCs, by releasing paracrine substances, can assist the survival of the surrounding tissue (Arthur, Gronthos 2020).

## Biomaterials in bone tissue engineering

BTE aims to produce implantable grafts that leverage the natural regeneration capacity of bone to treat trauma, cancers, and other bone diseases. The physical microenvironment provided by scaffolds closely resembles the native 3D microenvironment encountered by bone cells (Paladini, Pollini 2022). The constituent of biomaterials and their assembly technique determine attributes of scaffolds, including mechanical abilities, geometry, and porosity structural features. These features can be modified to regulate its osteogenic potential. For example, it has been demonstrated that while smaller pores improve in vitro osteogenesis (Nikolova, Chavali 2019). Ideal bone grafting materials should have good biocompatiblity, osteoconductivity, osteoinductivity, biodegradability and mechanical characteristic similar to bone (El-Rashidy et al. 2017).

Recent research has generated in vitro models for implantable bone grafts using human mesenchymal stem cells (hMSCs) with specific biomaterials (scaffolds) and stimuli like growth and differentiation agents. Numerous studies demonstrate the ability of hMSCs isolated from adipose tissue or bone marrow to stimulate bone repair when combined with biomaterials (De Wildt et al. 2023). Biomaterials are usually solid, rigid or porous and include ceramic, polymer materials and their composites that can be used to develop by operating as extracellular matrix models (Mazzoni et al. 2023) and each with their respective advantages and disadvantages (Table 1).

**Table 1:** Types of Biomaterials with their advantages and disadvantages

Types of Biomaterials	Examples	Advantages	Disadvantages
**Ceramics**	Hydroxyapatite (HA)	Biocompatibility \ Mimics bone tissue composition \ Osteogenic properties	Brittleness makes it prone to cracking under mechanical load \ Poor tensile and shear strength
	Tricalcium phosphate (TCP)	Promotes osteointegration \ Biodegradable, supports natural bone regeneration	Rapid degradation in highly acidic environments \ Mechanical integrity decreases over time
	Biphasic calcium phosphate (CaP)	Combines the properties of HA and TCP \ Good chemical stability and mechanical resistance	Variable degradation depending on composition
**Natural Polymers**	Collagen (Coll)	Biocompatibility \ Promotes tissue regeneration \ Resembles ECM	Poor mechanical resistance \ Rapid degradation reduces long-term structural stability
	Chitosan (CS)	Antibacterial properties \ Porosity supports cell growth \ Promotes neovascularization and cell proliferation	Low mechanical strength \ Limited solubility in neutral or basic pH environments
	Alginate (Alg)	Porous structure stimulates vascularization, adhesion, and cell proliferation \ Supports oxygenation and cell migration	Poor mechanical properties require blending with stronger materials \ Limited cell adhesion without functionalization
	Hyaluronic acid (Hay)	Stimulates angiogenesis \ Promotes rapid MSC differentiation \ Enhances bone formation	High cost limits large-scale applications \ Fast degradation in vivo without crosslinking with other materials
**Synthetic Polymers**	Poly-ε-caprolactone (PCL)	Slow and controllable degradation ideal for long-term scaffolds \ Good processability for custom shapes and porosities	Slow degradation can delay tissue regeneration \ Poor mechanical strength under dynamic loads
	Polylactic acid (PLA)	High mechanical strength \ Biodegradability \ Easy to process into fibers, films, or 3D structures	Acidic degradation byproducts that alter local pH, may causes inflammation of surrounding tissues
	Polyglycolic acid (PGA)	Rapid biodegradation promotes fast replacement by natural tissues \ High biocompatibility	Rapid degradation compromises mechanical stability
	PLGA copolymer	Improved osteoconduction compared to single PLA or PGA \ n- Versatile PLA/PGA ratios to adjust properties	Degradation generates acidic byproducts similar to PLA and PGA, potentially affecting the surrounding microenvironment
	Polyvinyl alcohol (PVA)	High water solubility for easy processing \ Excellent mechanical properties when crosslinked	Poor biocompatibility without chemical modification \ Requires crosslinking to achieve adequate mechanical strength
**Ceramic-Polymer Composites**	Hydroxyapatite / Collagen (HA / Coll)	Excellent osteoinductivity and biocompatibility \ Immunomodulatory potential \ Promotes MSC proliferation and differentiation	Despite the ceramic-polymer combination, it lacks sufficient strength for applications in areas subjected to high mechanical stress

The greatest advantage of ceramics is the biocompatibility with the human body. Hydroxylapatite (HA) and tricalcium phosphate (TCP) are popular ceramic biomaterials due to their osteogenic qualities and ability to connect with host bone. A common synthetic ceramic called “biphasic calcium phosphate” is made by combining TCP and HA in varying ratios to enhance the characteristics of each mineral (Bharadwaz, Jayasuriya 2020). Research has demonstrated that cation addition (strontium Sr^2+^ and magnesium Mg^2+^) in calcium phosphate (CaP) based biomaterials modify chemical-physical properties like microstructure, solubility, and crystallinity as well as improving mechanical qualities (Tarafder et al. 2013). In particular, Mg ^2+^ stimulates the creation of new bone mineral nuclei, consequently stimulating the growth of new tissue. Montesi et al. described a strontium-doped cement HA enriched with sodium alginate and showed that Sr^2+^ may lead to osteogenic differentiation at different dosages (Montesi et al. 2017).

Polymer-based biomaterials can be either synthetic or natural. Natural polymers, like collagen (Coll), chitosan (CS), alginate (Alg), and hyaluronic acid (Hay) are used in bone regeneration due to their biocompatibility and resemblance to the ECM (Bharadwaz e Jayasuriya 2020). However, pure collagen lacks mechanical strength and is therefore often combined with ceramic biomaterials. CS is a linear polymer produced by chitin’s deacetylation. CS is ideal for bone repair due to its antibacterial properties, porous structure suitable for cell growth, and ability to stimulate osteoblast and mesenchymal cell proliferation and neovascularization in vivo (Guo et al. 2021)*.* Alg is a marine polysaccharide composed by glucuronic and d-mannuronic acids residues, which can be used as a bone substitute since its porous structure stimulates vascularization, oxygenation, cell migration, adhesion, and proliferation (Valente et al. 2012). According to reports, high-molecular-weight hyaluronic acid (1900 kDa) significantly promotes bone formation by stimulating angiogenesis and faster MSC differentiation in injured bone region (Zhai et al. 2020).

Synthetic polymers, like poly(ε-caprolactone) (PCL), polylactic acid (PLA), polyglyclide (PGA), poly-(DL-lacto-co-glycolic-acid) copolymer (PLGA), polyvinyl alcohol (PVA), and polyvinylpyrrolidone (PVP), are useful in BTE. PCL is FDA-approved, highly biocompatible, and widely used (Iaquinta et al. 2019). Because PLA and PGA have limited compressive strength and osteoconductivity, they are not ideal as biomaterials for bone tissue reconstruction. Osteoconductivity and solubility are enhanced in PLGA copolymers containing different ratios of PLA and PGA (Gentile et al. 2014). However, the use of synthetic polymers has limitations. Acid products are generated by their deterioration process, which might influence the local "microenvironment" and change the pH locally (Iaquinta et al. 2019).

Composite biomaterials are created through the integration of ceramic scaffolds with polymers. Due to its great biocompatibility and mechanical hardness, this kind of biomaterial is well-suited for tissue engineering applications (Niemeyer et al. 2004). Collagen and hydroxylapatite-based composites (COLL/HA) are particularly effective for bone transplantation. Research on hBMSCs has shown excellent osteoinductivity and biocompatibility for COLL/HA scaffolds like Coll/Pro Osteon200, combining hydroxyapatite (Pro Osteon 200) and collagen (Avitene) (Mazzoni et al. 2020; 2021). Studies on HA (Bio-Oss) and collagen (Avitene) composites have revealed their immunomodulatory potential and ability to promote hADSC proliferation and differentiation (Iaquinta et al. 2022).

Several scaffolds are also frequently used as drug delivery systems. The multifunctional features of COLL/HA composite materials can be induced by incorporating various components, such as bone morphogenic protein (Lee et al. 2021), bisphosphonates, antibiotics (Filip, Mocanu 2022) and anticancer drugs (Lanzillotti et al. 2024). The most studied drug-delivery systems are COLL/HA loaded with antibiotics or analgesics, after surgical intervention for the treatment of severe bone defects (Filip, Mocanu 2022). Bone morphogenic protein incorporation in composites biomaterials improves bone regeneration process; bisphosphonate indirectly favors bone formation by suppressing bone resorption (Lee et al. 2021). In bone cancer, tumor mass resection is often required, leaving a bone defect that needs to be filled with bone-regenerative material. Bone fillers can incorporate pharmaceutically active substances such as antitumoral drugs, releasing them in situ. In a recent study, Strontium-doped apatitic bone scaffolds used as carriers for methotrexate (MTX) or doxorubicin (DOX) demonstrated cytotoxic and pro-apoptotic activity against human osteosarcoma cell lines (Lanzillotti et al. 2024).

In general, biomaterials are designed to be solid, rigid, or porous, often used in applications where a stable and resistant structure is needed. For example, synthetic polymers are processed to create scaffolds or rigid implants, suitable for supporting hard tissues like bones. Ceramics, such as hydroxyapatite and calcium phosphates, provide a solid, crystalline structure, ideal for replicating the rigidity of bone tissue and promoting integration with natural tissue. In solid biomaterials, the final structure is designed to withstand mechanical loads, with porosity that facilitates cellular infiltration and tissue regeneration without sacrificing rigidity (Suamte et al. 2023). They are primarily employed in contexts requiring mechanical support or the replacement of rigid structures, such as for prostheses and bone scaffolds (Suamte et al. 2023).

These same ceramic, polymeric and composite matrices can be structured in a completely different way to form hydrogels. These are soft and elastic materials made up of three-dimensional networks of hydrophilic polymers that retain large amounts of water, often up to 99% of their weight (Sánchez-Cid et al. 2022). Polymers such as collagen, alginate, or hyaluronic acid, also used in solid biomaterials, are modified here to form a highly hydrated and flexible structure. Ceramics like hydroxyapatite can also be incorporated into hydrogels, but in dispersed form, to enhance bioactivity without compromising the softness of the structure (Zhao et al. 2023). This approach allows hydrogels to simulate a natural biological environment, promoting cell growth, nutrient diffusion, and the transport of biochemical signals. Hydrogels are used for purposes that require a more delicate biological interaction. Thanks to their ability to mimic the extracellular matrix, they are widely employed in the construction of three-dimensional models, such as spheroids and organoids, and in controlled drug delivery systems (Yue et al. 2020). Spheroids, which are three-dimensional cellular aggregates, benefit from the use of hydrogels because these materials allow for the diffusion of nutrients, oxygen, and biochemical signals essential for cell survival and differentiation. Organoids, which represent more complex and organized models of tissues or miniature organs, require an environment similar to the extracellular matrix to develop, efficiently provided by hydrogels (Yue et al. 2020). Another type of structure widely used for the creation of three-dimensional cell models, particularly spheroids, is biomaterial-based films (biofilms). These are thin layers of biocompatible biomaterials made from polymers or a combination of polymers and ceramics, as mentioned above. Biomaterial-based films provide an initial surface for cell aggregation and spheroid formation. The structure of the films is typically thin and uniform, but it can be designed to have controlled porosity to allow the passage of nutrients, gases, or bioactive molecules (Lu et al. 2017).

## Transwell and co-culture bone cells

Cell morphology, molecular processes, and differentiation patterns can be studied using single-cell type cultures. However, in these systems, the signals between various cell types are completely unidentifiable. Co-culture models more closely resemble the in vivo environment because interaction between different cell types leads to the transmission of intercellular signals through junctions, exosomes, and their autocrine/paracrine activities (Borciani et al. 2020). In vitro co-culture methods can be considered a potent instrument for improving our comprehension of the tight cellular interactions which take place in physical contact and/or soluble chemicals (Borciani et al. 2020).

Challenges of co-culture system include selecting parameters for the coexistence of two or more distinct cell types: cell relationship, shared culture mediums, working time, and tools that can distinguish between different cell isotypes (Esmaeili et al. 2024).

Two distinct approaches can be used to create a 2D co-culture, with a direct or indirect physical contact system. Physical interactions and autocrine/paracrine signals can be examined in direct contact co-culture. However, a drawback of this approach is the complexity in fully understanding the different contributions made by various kinds of cells combined in the same environment (Borciani et al. 2020).

In indirect cell co-cultures, the various cell types are physically separated by a transwell porous membrane, by which the culture medium and other molecules can flow from one vessel to another one. With the release of proteins, extracellular vesicles, and soluble factors from one cell type influence another cell type through paracrine signaling. Such information sharing has a significant impact (Im 2014).

Taylor and collaborators co-cultured osteoblasts and osteocytes in transwell (Taylor et al. 2007). The results demonstrate the critical role that gap-junction intercellular communication plays in the osteoblastic response to mechanical stimuli given by osteocytes. Suda et al. emphasized the necessity of osteoblast and osteoclasts collaboration, because the development of Tartrate-resistant acid phosphatase (TRAP) mononucleate cells requires the presence of osteoblastic cells or other inducers (Takahashi et al. 1991). Chen et al. investigated the angiogenic and osteogenic effects of co-culture on calcium phosphate cement (CPC) using MSCs derived from various sources and human umbilical vein endothelial cells (hUVECs). The research data showed superior osteogenic and angiogenic potential in co-cultures compared to individual cultures in the CPC scaffold (Chen et al. 2018). On Zirconia Toughened Alumina** (**ZTA) ceramics, Halai et al. have successfully produced a novel human co-culture of BMSC and bone marrow hematopoietic cell (BMHC). After 21 days of growth on micropatterned surfaces, BMSC cells were able to generate bone nodules (Halai et al. 2014). The Silverwood et al. research of co-cultured osteoblasts and osteoclast on titanium material surfaces reveals a decrease in osteoclastogenesis and an increase in gene expression linked to osteoblastogenesis (Silverwood et al. 2016).

## Bone spheroids

A promising 3D approach is the use of spheroids, which offer a tissue-like physiochemical environment in vitro. Spheroids can be a useful model for studying bones and their biological processes due to the high production of ECM and cell-ECM interactions (Gionet-Gonzales, Leach 2018), which offer mechanical forces and biomechanical signals that regulates gene expression, proliferation, differentiation, and helps signal transduction between the cytoskeleton and the extracellular environment (Boscaro, Sikorski 2024).

The aggregate formation of spheroids is similar to the natural assembly process of embryogenesis, morphogenesis, and organogenesis. Cell–cell and cell-ECM interactions mediated by cadherins and integrins are essential for the creation and preservation of the cell aggregate (Cui et al. 2017). There are multiple steps in the development of a spheroid. First, individual cells in the suspension clump together to create loosely anchored cell spheroids. Preliminary aggregation is promoted by ECM fibers, which include binding of the peripheral cell surface to integrin. Next, E-cadherin stimulates robust adhesion of the initial cell aggregation, by forming homophilic interaction between peripheral cell cadherins. Furthermore, β-catenin promotes the transmission of cellular signals. Actin facilitates interactions among neighboring cells, influencing agglomeration and stemness. Strongly sticky multicellular spheroids are the final result (Tsai et al. 2015).

**Fig. 5:**
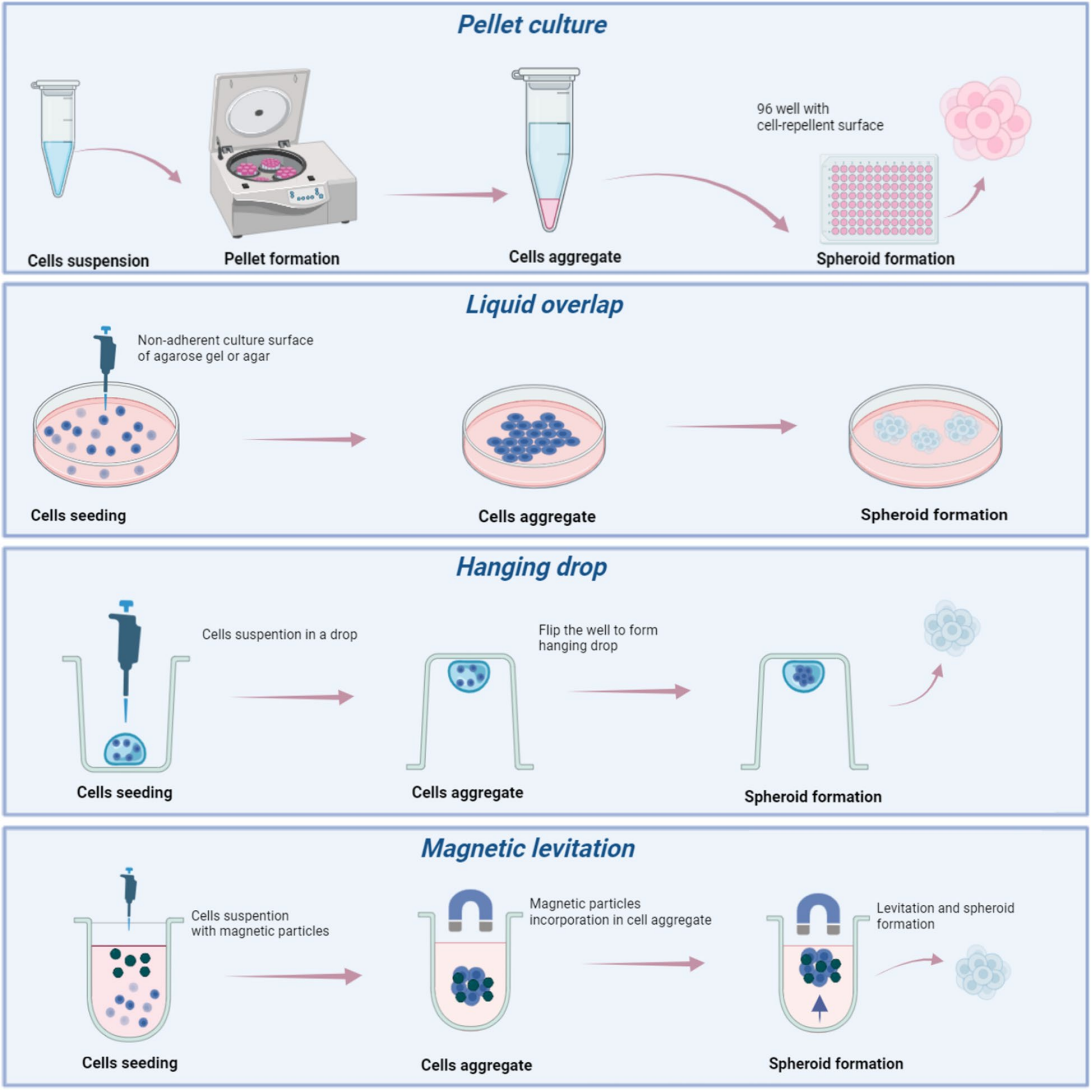
Techniques used for spheroids generation. In the pellet culture technique centrifugal force concentrates the cells on the bottom of the tube. The proximity of the single cells at the tube bottom maximizes cell–cell adhesions. After the cell pellets are resuspended in spheroid formation cell culture medium. Cells in the medium are added to a 96-well U-bottom plate with a cell-repellent surface. Liquid overlay culture technique, also called “static suspension culture”, forms spheroids by interrupting the adhesion of cells on non-adherent culture plates. Agarose gel or agar substrate is commonly used to create non-adherent culture layers. By encouraging cell–cell adhesive molecules, cells naturally form spheroids above the non-adherent surface. Droplet-shaped spheroids are generated using the hanging drop culture technique, which efficiently generates specific size spheroids. To achieve appropriate cell density, technique begins with a monolayer cell culture, from which cells are prepared as a suspension with culture media. The cell suspension is then pipetted into wells of a mini plate. After it is completely upside-down. Surface tension keeps the cell suspension drops fixed to the mini plate on the inverted surface. This approach uses the simultaneous action of surface tension and gravitational force to create spheroids as droplets. Magnetic particles are used in magnetic levitation-based culture. During cell growth, cells are combined with magnetic particles and exposed to magnetic force. Cells remain levitation against gravity. This state causes a change in the mass and cell’s shape and encourages cell–cell contact, resulting in cell aggregation

Challenges include angiogenesis and nutrient absorption. Cells in the inner regions do not receive continuous nutrients and oxygen inputs like the cells outside. Moreover, because of diffusion to the inside structure becomes more difficult with increasing aggregate size, the size control is one of the most crucial element (Laschke, Menger 2017). Another crucial factor is the spheroid environment, which influences the characteristics of spheroids depending on materials, molecules, or stimuli used (Decarli et al. 2021). Various technical methods are used for spheroid formation: (a) Pellet culture, (b) Liquid overlap, (c) Hanging Drop and (d) Magnetic levitation (Fig. 5).

In the pellet culture technique, centrifugal force concentrates the cells on the bottom of the tube. The proximity of the single cells at the tube bottom maximizes cell–cell adhesions. After the cell pellets are re-suspended in spheroid formation cell culture medium in a 96-well U-bottom plate with a cell-repellent surface (Maritan et al. 2017). Kengo Iwasaki et al. reported that periodontal ligament stem cells (PDLSCs) in pellet culture develop spontaneously spheroid masses. When compared to 2D culture, spheroid culture significantly increased the expression of genes related to angiogenesis and anti-inflammatory responses (Iwasaki et al. 2019).

Liquid overlay culture technique, also called “static suspension culture”, forms spheroids by interrupting the adhesion of cells on non-adherent culture plates. Agarose gel or agar substrate is commonly used to create non-adherent culture layers. By encouraging cell–cell adhesive molecules, cells naturally form spheroids above the non-adherent surface (Costa et al. 2018).

Droplet-shaped spheroids are generated using the hanging drop culture technique, which efficiently generates specific size spheroids. The cell suspension is pipetted into wells of a mini plate, that is completely upside-down. Surface tension keeps the cell suspension drops fixed to the mini plate on the inverted surface. This approach uses the simultaneous action of surface tension and gravitational force to create spheroids as droplets (Achilli et al. 2012). Murphy et al. realized MSC spheroids employing the hanging drop technique with an increasing number of cells. The smallest spheroids showed excellent cells proliferation and metabolic activity, without the activation of apoptotic process. Due to their strong proangiogenic ability and resistance to apoptosis, MSC spheroids have a high potential for osteogenesis (Murphy et al. 2014).

Magnetic particles are used in magnetic levitation-based culture. During cell growth, cells are combined with magnetic particles and exposed to magnetic forces (Kronemberger et al. 2020). Cells remain levitation against gravity. This state causes a change in the mass and cell’s shape and encourages cell–cell contact, resulting in cell aggregation (Kronemberger et al. 2020). Generation of 3D human fetal osteoblast (hFOB) spheroids using magnetic levitation was the goal of a recent study conducted by Iñigo Gaitán-Salvatella. The results suggest that magnetic levitation culture stimulate the growth of 3D stable osteoblast spheroids and provide a viable approach to generate a 3D construct for bone regeneration and mineralization (Gaitán-Salvatella et al. 2021).

Spheroids are frequently implanted into biomaterial to stabilize aggregate and shape. Hydrogels, biofilms, and particles are examples of support structures used in spheroid production, as described in the chapter "Biomaterials in Bone Tissue Engineering".

Hydrogels are ideal for encapsulating spheroids due to ECM-like characteristics. With this technique, cellular apoptosis is reduced and cell viability is successfully increased (Kim et al. 2019). In addition, compared to the non-entrapped cells in the monolayer culture, the osteogenic differentiation potential is constantly maintained and proangiogenic factor secretion is stimulated in the hydrogel-entrapped cells. Hydrogels with ECM-like physicochemical and biomimetic characteristics may offer functional niches that support wound healing and have the ability to self-renewal (Ho et al. 2016).

The study by Kazuhide Mineda et al. demonstrates the therapeutic potential of human ASCs spheroids produced in hyaluronic acid hydrogels. The results highlight the spheroids' remarkable ability to secrete growth factors and promote tissue regeneration (Mineda et al. 2015). Jacklyn Whitehead et al. show that dynamic mechanical features of viscoelastic alginate improve the therapeutic potential of MSC spheroids for bone formation and repair (Whitehead et al. 2021).

Biomaterial-based films (biofilm) can be created using a variety of techniques, including stamping and photolithography. The films' component ratios play a crucial role in cellular adhesion and proliferation, as well as the size and rate of spheroid formation. Culture on film leads to increased expression of differentiation markers in stem cells (Lu et al. 2017). Chitosan is one of the biomaterials employed in these films. A study by Nai-Chen Cheng et al. reports that ASCs cultured on chitosan films demonstrate increased regeneration potential during spheroid formation (Cheng et al. 2013).

Particulate factors have been employed to regulate the microenvironment and solve the insufficient amount of nutrients and oxygen in the spheroid. Particles can control the environment inside spheroids, with the aim of increasing cell viability and proliferation (Kim et al. 2018). On the other hand, particles inhibit internal connection between spheroids' adjacent cells and this promote a non-uniform cell differentiation (Abbasi et al. 2018). The use of particles that release differentiation and growth factor in the spheroid microenvironment, which allows spatial control of differentiation, is the solution. Research has shown that excellent osteogenic differentiation might be induced in particles encapsulating MSCs (Chan et al. 2010).

## Spheroid-on-chip for bone research

Microfluidic technology and micro-nano fabrication have emerged as powerful tools in the spheroids formation (Wang et al. 2020). These chips are miniaturized devices with precise control of fluid flow at the microscale level over the spatial distribution and temporal dynamics of spheroids. Thanks to the chip's microscale dimensions, researchers can manipulate media, nutrients, and cells to create spheroids with controlled growth and formation. Spatial–temporal control also permits the creation of complex architectures and makes it easier to study cell–cell interactions (Fang et al. 2021).

In osteogenesis, a dynamic flow of media mimicking in vivo vascularization is crucial, not only for continuous perfusion of nutrients and removal of cellular waste metabolites, but also for inducing fluid mechanical stimulation, mineralization and ECM deposition, which leads to bone regeneration. Notably, the flow of media exposes the cells to shear stress similar to the dynamic niche found in bone cell physiology (Bhaskar et al. 2018).

The use of microfluidic chips to control the development of dynamic cell cultures is a relatively recent application that shows improvements in 3D bone cell spheroid formation. In a recent study, a bone-on-a-chip murine spheroidal 3D cell culture showed increased mineralization and viability compared to static conditions. This successfully confirms the beneficial effects of a dynamic culture environment on osteogenesis. The experiment was repeated with the same results in spheroids produced from primary human pre-osteoblasts (Killinger et al. 2023). Additionally, microfluidic chips can be coupled with biosensors and analytical techniques to measure various parameters, including oxygen levels. An oxygen-permeable culture chip (Oxy chip) can be used to form MSCs spheroids. The use of the Oxy chip avoids hypoxia in the spheroid nucleus and improves osteoblastic differentiation of MSCs, compared to conventional spheroid culture methods (Sato et al. 2019).

## Bone organoids

Organoids are 3D cell culture systems that self-organize into structures that mimic tissues and organs, formed by stem cells or organ progenitors. They are widely used in studies of differentiation, drug development, pharmacological applications, cancer research, investigations of gene and protein expression (Ravi et al. 2015).

Bone organoids are an advantageous model for studying bone regeneration mechanisms because they imitate the spatial organization and functions of bone tissues. Stem cells, matrix scaffolds, and mechanical stimulation are fundamental choices to guarantee the growth and differentiation of cellular bone organoids (Millan et al. 2018). Osteoblasts, osteoclasts, and stem cells are used in bone organoids. Co-culturing multiple cell types in bone organoids better replicates the physiology and pathology of human bone tissue, including self-renewal, migration, and differentiation processes (Chen et al. 2022).

Organoids derived from iPSCs are realized in accordance with the developmental process. As a result, iPSC organoids are frequently in the embryonic stage and are more suited for researching the biology and physiology of early organs (Huang et al. 2023). Despite the various challenges involved in creating bone organoids, a large number of iPSC-derived bone organoids have been created for bone repair. O'Connor et al. used mice iPSCs to create bone analogues to investigate the link between bone and cartilage. TGF-β3 and BMP2 were injected sequentially in chronological order to encourage the development of single cells into organoids and osteogenesis process (O’Connor et al. 2021).

ASCs usually differentiate into particular tissue or organ cells and produce organoids that are made up of one kind of cell. (Huang et al. 2023). BM-MSCs are extensively employed to generate bone organoids. Scotti et al. use a collagen scaffold with BM-MSC to generate endochondral osteogenesis organoid model. The culture media was then supplemented with IL-1β to encourage tissue reconstruction. The BMSC-based organoid can conduct physiological processes such as angiogenesis, hematopoiesis, and bone repair (Scotti et al. 2013).

**Fig. 6:**
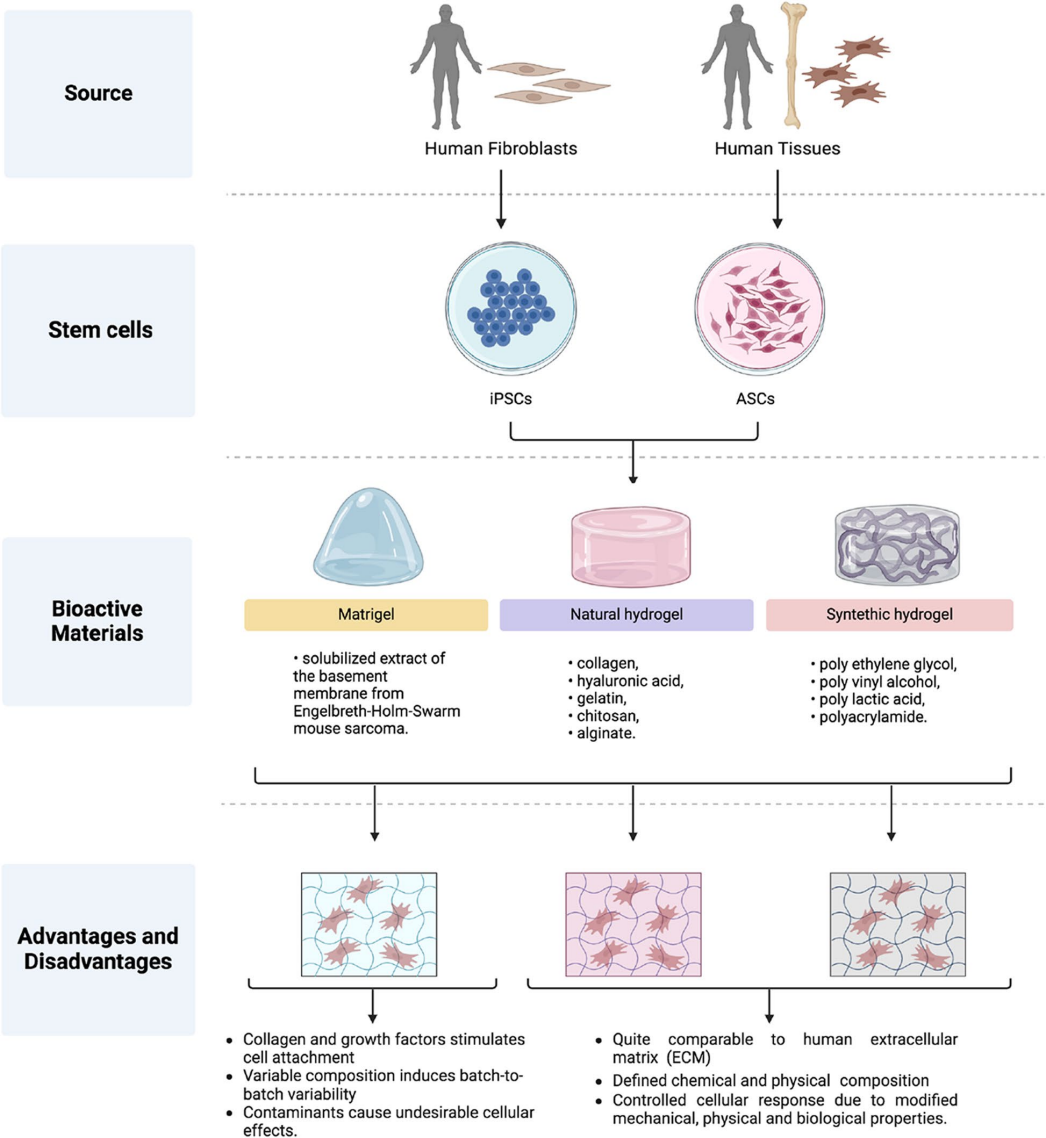
Main components of bone organoid production. Bone organoids, derived from stem cells, allow studying how cell–cell and cell-ECM interactions in 3D affect bone differentiation. iPSC organoids are suitable for researching primitive organ biology and physiology. iPSCs are generated by reprogramming fibroblasts with nuclear transcription factors, then adding osteoblast-specific factors for osteogenic differentiation. ASCs, like bone marrow or adipose-derived MSCs, produce single-cell type organoids without needing different differentiation conditions. Bioactive materials, like Matrigel and hydrogels, are key components**.** Matrigel is a solubilized extract of the basement membrane from Engelbreth-Holm-Swarm mouse sarcoma. Its high levels of collagen and growth factors promote cell adhesion. However, its application is restricted in various contexts because it originates from animals, has unpredictable composition, may be contaminated with xenobiotic contamination and exhibits batch-to-batch variability. Hydrogel is made up of polymers, which can be either natural or synthetic, and can be tailored to have specific chemical and physical properties, creating an optimized environment for cell culture. Its modifiable mechanical, physical, and biological characteristics enable a controlled response from cells. Natural hydrogels closely resemble the extracellular matrix (ECM) because they contain its primary components. Common examples of natural hydrogels include polysaccharides and proteins like collagen, hyaluronic acid, gelatin, chitosan, and alginate. On the other hand, synthetic hydrogels are composed of hydrophilic polymers such as polyethylene glycol, polyvinyl alcohol, polylactic acid, and polyacrylamide

Bioactive materials are a key component in the production of stable bone organoids (Li et al. 2023) (Fig. 6). Matrigel is a solubilized extract of the basement membrane from Engelbreth-Holm-Swarm mouse sarcoma (Passaniti et al. 2022). Matrigel is one of the primary tools used to culture organoids in the laboratory, providing a biological and three-dimensional base that simulates the natural extracellular matrix. It contains many growth factors, including collagen IV, entactin, and laminin (C. Li et al. 2023), that stimulates cell attachment and proliferation. However, its use is limited in several applications due to its animal-derived nature and batch-to-batch variability (Huang et al. 2020). Chao Li et al. show how Matrigel for bone organoids have physico-chemical properties closer to the real human ECM, but uncontrollable factors of batch-to-batch stability limit their use (Li et al. 2023).

Hydrogel, is a promising alternative that offers a better-regulated environment for cell culture (Blanco‐Fernandez et al. 2021). Hydrogel can be designed to possess specific chemical and physical characteristics and offer a better-regulated environment for cell culture (Blanco‐Fernandez et al. 2021). It is composed of polymers, either natural or synthetic, as described in the chapter "Biomaterials in Bone Tissue Engineering". Polysaccharides and proteins such as collagen, gelatin, chitosan, and alginate are classic natural hydrogels. Pure collagen's limited mechanical strength has been improved by combining it with other biomaterials like graphene oxide (GO) nanosheets, PLGA, HA, and CaP (Wu et al. 2023). Gelatin is a hydrocolloid with excellent biocompatibility and biodegradability. Its low mechanical modulus and fast degradation can be improved by substituting its amino groups with methacryloyl units, creating gelatin methacrylate (GelMA) building blocks. Liu et al. demonstrated that GelMA/struvite composite hydrogel with DPSCs organoid showed excellent cell viability and osteogenic properties (Liu et al. 2015). The biodegradability, biocompatibility, and antibacterial activity of CS derivative hydrogels have made them a viable support for organoids (Pan et al. 2021). Raftery et al. loaded CS nanoparticles with osteogenic (BMP-2) and angiogenic (VEGF) genes, to create a novel gene-activated hydrogel. According to results, MSC protein expression in the hydrogel platform persisted for 28 days, promoting MSC osteogenesis (Raftery et al. 2017.) Alginate's advancement in bone tissue engineering is limited by its inadequate mechanical characteristics. Adding metal, bioactive glass, and HA functional nanoparticles to alginate hydrogels is the solution. For example, Piyachat et al. (Chuysinuan et al. 2021) created an injectable hydrogel of HA-compound fibroin-Alg to improve bone healing.

Hydrophilic polymers such as poly (ethylene glycol), poly (vinyl alcohol), poly (lactic acid), and polyacrylamide compose synthetic hydrogels. Vallmajo-Martin et al. (Vallmajo‐Martin et al. 2020) combined hyaluronic acid and polyethylene glycol hydrogels to generate bone marrow organoids. These hybrid hydrogels induced the proliferation and differentiation of bone marrow mesenchymal stem cells (BMSCs) and hematopoietic stem cells (HSCs) (Vallmajo‐Martin et al. 2020).

Research on bone organoids is still in its early stages but has made significant progress thanks to the use of stem cells, particularly iPSCs and MSCs. These approaches aim to create bone organoids that replicate the native architecture of bone and possess functional attributes such as the ability to integrate with host tissues and support hematopoiesis. In 2018, Perez et al. used iPSCs to produce bone implants capable of promoting fracture repair by inducing osteoblast differentiation, a significant advancement for addressing delayed healing and segmental bone defects (Perez et al. 2018). Later, in 2020, osteochondral organoids were realized from mouse iPSCs to study cartilage-bone interactions (O’Connor et al. 2021), while in 2022 Frenz et al. (Frenz et al. 2022) developed bone organoids derived from hiPSCs, demonstrating their potential for studying bone diseases. In 2024, Frenz-Wiessner et al. generated bone marrow-like organoids (BMOs) from iPSC capable of supporting lifelong hematopoiesis, a breakthrough that could revolutionize the understanding of bone and joint diseases (Frenz-Wiessner et al. 2024). In parallel, the use of MSCs has led to significant advancements in building bone organoid models. In 2021, Akiva and colleagues created the first functional 3D in vitro model to study early bone formation by cultivating BMSCs on silk fibroin scaffolds and exposing them to mechanical stimulation in a bioreactor (Akiva et al. 2021). This approach enabled the formation of an early-stage bone organoid resembling woven bone. In 2023, it has been demonstrated that co-culturing BMSCs with hematopoietic cells could support the growth of the latter in bone marrow organoid models (Khan et al. 2023). Recently, it has been established that the addition of a basement membrane extract enhances the endochondral ossification process in osteochondral organoids generated from MSCs (Notoh et al. 2024).

Recent developments have also led to the creation of bone organoids to simulate specific physiological and pathological processes. For instance, callus bone organoids, designed using hydrogel microspheres loaded with BMSCs, successfully regenerated bone within four weeks, as reported (Xie et al. 2022). Similarly, trabecular bone organoids have been developed to study diseases such as osteoarthritis (Park et al. 2021) and to address issues related to osteoporosis and low bone density (Iordachescu et al. 2021). Additionally, tumor organoids, which retain the genetic and phenotypic characteristics of the original tumor, have been applied in the study of bone tumors, such as osteosarcoma, using organoid models and next-generation sequencing, as reported (Rausch et al. 2023).

Despite their potential, bone organoids still face significant limitations. The complexity of bone tissue, which includes the circulatory system, extracellular matrix, and various cell types, is difficult to fully replicate in current models. Moreover, the production of organoids lacks standardization, involving many variables such as the cell source, culture medium components, and materials for 3D scaffolds. Another crucial challenge is vascularization: without a vascular network, organoids cannot provide adequate nutrients to cells (He et al. 2022). Looking to the future, scaffold-free culture methods, such as bone micro-organoids, could be enhanced to address the difficulty of mimicking a complex system. These methods represent an extension of 3D printing technology with promising applications. Additionally, the adoption of consistent policies and standards could improve the reproducibility of the models. Innovative solutions to poor vascularization include microfluidic systems, engineered vascularization, co-culture with endothelial cells, or the stimulation of angiogenesis through VEGF and FGF.

## Organ-on-chip for bone research

Microfluidic-based organ-on-chips incorporate miniaturized cell-culturing microenvironments with microchannels and compartments, which replicate the natural environment of human cells by combining tissue engineering, microfluidics, and lab-on-a-chip technologies. Microfluidic technology improves organoid research in three aspects: easier control of microenvironment, construction of multiorgan systems, and lower variability of parallel experiments (Syahruddin et al. 2023).

Recently, organ-on-a-chip enabled researchers to study bone physiology and lead to the creation of the bone-on-a-chip (BoC) device (Nasello et al. 2021).

BoC devices provide an in-depth view of bone development, support therapies for bone-related diseases such as osteoporosis and bone metastasis, and simulate cellular interactions, mechanical stimuli, innervation, and vascularization. Pioneering studies have developed biomimetic platforms to replicate pathological conditions such as osteonecrosis, osteoporosis, and fracture healing (George et al. 2018). Sheyn et al. developed a bone-on-a-chip system with constant flow, compared to static culture, to evaluate cell survival and osteogenic differentiation through gene expression analysis, and immunostaining for osteogenic markers (Sheyn et al. 2019).

The BoC devices could improve the automation and throughput of studies investigating the cell–cell interaction in bone remodeling. In 2017, Middleton and colleagues have successfully cultured osteocytes and osteoclast precursors within a microfluidic co-culture system, to examine osteoclast precursor responses to signals produced by osteocytes, as well as osteoclast modulation by osteocyte signals (Middleton et al. 2017). Moreover, Nasello et al. in 2020 set-up a system to mimic osteoblast development into osteocytes using primary human osteoblast seeded in type I collagen hydrogel with modified cell densities (Nasello et al. 2020). The results of this study showed that cell densities applied within bone-on-a-chip affect the proliferation, alkaline phosphatase (ALP) activity, and production of osteocyte or osteoblast specific markers (Nasello et al. 2020). Yvanoff et al. in 2022, created microarrays composed of osteoblasts and osteocytes within microfluidic chips using non-contact robotic printing and microfluidic techniques. This BoC device allowed osteoblasts and osteocytes to communicate at localized interfaces under fluid flow. The authors verified the calcium-induced response in the osteocyte-osteoblast network following mechanical stimulation (Yvanoff, Willaert 2022). Interestingly, several approaches have been developed to investigate the role of innervation in skeletal development. To this purpose, Silva et al. established a microfluidic device to study the impact of dorsal root ganglion (DRG) neurons on the capacity of MSCs to differentiate into osteoblasts. Their results showed that the direct interaction between DRG neurons and MSCs increased the differentiation of MSCs into osteoblast (Silva et al. 2017).

Bone is a highly vascularized tissue and bone angiogenesis has important roles in endochondral bone formation and repair. Bertassoni et al. developed microchannel networks within GelMA using 3D micromolding, promoting microvascular formation and enhancing the viability and osteogenic differentiation of MC3T3 cells (Bertassoni et al. 2014). Jeon et al. created a microvascularized bone-mimicking device using a hydrogel containing MSCs, osteoblasts, and HUVECs, demonstrating the formation of functional vascular networks with specific vascular markers (Jeon et al. 2015). Moreover, Glaser et al. in 2022 (Glaser et al. 2022) presented a bone marrow-on-a-chip model, including perivascular and endosteal niches complete with dynamic, perfusable vascular networks, by using endothelial colony-forming cells and BMSCs.

BoC devices were also developed to study bone disease. Paek et al. in 2023 created a BoC platform with osteocytes embedded in a decellularized matrix and surrounding osteoblasts, simulating the osteon and reflecting bone remodeling in vivo. The model demonstrated efficacy in testing an anti-sclerostin drug for osteoporosis, supported by an AI-based analysis system (Paek et al. 2023).

The BoC models offer great potential in translational research, but there are several challenges to address. A major issue is the incomplete representation of the biological processes of bone, as current models fail to fully replicate the bone environment, which includes numerous cell types. Additionally, another challenge is isolating and co-cultivating different types of bone cells in a single device. For example, osteoblasts and osteoclasts come from different tissues and have distinct proliferation and differentiation behaviors, which complicates their simultaneous cultivation. Moreover, the dynamic process of mineralization and demineralization has not been fully replicated in BoC models. The difficulty in introducing a mineralized environment and reproducing bone resorption through osteoclasts is one of the main current limitations. Another problematic aspect concerns biosensors, which often lack sufficient sensitivity to measure the low concentrations of biomarkers, making it challenging to accurately monitor cellular processes in real-time.

The future prospects for Bone-on-a-chip (BoC) models focus on improving their ability to replicate the bone microenvironment, including all involved cell types, and integrating advanced technologies like highly sensitive biosensors. The goal is to develop models using patient-derived primary cells, enhancing treatment personalization. Challenges include overcoming issues in co-culturing bone cells, implementing controllable mechanical loading, and replicating dynamic bone processes. The integration of biosensors for real-time analysis and the use of materials that better mimic natural bone are also crucial. Finally, collaboration across scientific disciplines and large-scale production of biosensor-integrated BoC models are essential for both research and clinical applications.

## Conclusions

BTE approaches have been developed to improve the regeneration of bone defects and reduce morbidity, while ensuring the healing of damage. New BTE strategies must be evaluated in vitro for the ability to mimic in vivo bone repair process. Historically, in vitro 2D models have been used to analyze the physiological and pathological processes associated with bone. 3D cell cultures have demonstrated a range of advantages and innovations, including improved cell survival, comparable in vivo morphology and better interaction between cells and ECM. Recent studies have produced 3D in vitro models for implantable bone grafts that combine hMSCs and specific natural and synthetic biomaterials, revealing that hMSCs can promote bone repair when combined with biomaterials.

3D models based on spheroids and organoids make the study of cell–cell and cell–matrix interactions possible. The combined use of spheroids, various types of biomaterials and cells offers opportunities to study the field of bone tissue engineering. Understanding the biological mechanisms behind the creation of bone tissue in a spheroid-based model system requires a fundamental knowledge of cellular organization, extracellular matrix structure, and extracellular matrix mineralization.

3D bone organoids in vitro culture system highly simulates the in vivo precise position and spatial morphology of cells and matrix. There are promising potential applications of bone organoids for tissue engineering, however, there are still some challenges. Firstly, biomaterial design and directional differentiation of stem cells in bone organoids are still under investigation. Furthermore, bone organoids represent only one function for bone, such as bone formation, bone resorption or hematopoiesis. Achieving multifunctionality in an integrated bone organoid still represents a challenge.

Microfluidics is a new class of cell culture technology made possible by advancements in micro and nanoscale technology. Bone/cartilage organoid and spheroid on-chip systems have the huge potential to mimic the essential elements, biological functions, and pathophysiological response under real circumstances. Thus, they are important in vitro models to study bone regeneration and orthopedic diseases mechanism. The structure and function of bone is highly intricate and involves various cells, biochemical and biophysical factors. Therefore, future bone-on-chip models using bone organoids and spheroids must improve the control and precision of fluid-mechanical signals at the microscale, to obtain ever closer conditions to real human bone. Furthermore, the translation of microfluidic chip-based organoid and spheroid models into clinical applications requires validation and verification of their predictive capabilities.

Organoids and Organ-on-chip systems are transforming bone research, providing three-dimensional models that replicate the complexity of bone tissue and its biological processes. These technologies enable deeper insight into bone regeneration, cell–matrix interactions, and bone metabolism, proving essential for testing therapies, studying conditions such as osteoporosis and osteosarcoma, and developing biocompatible materials.

Despite their potential, several significant challenges remain. Among these are the difficulty of replicating functional vascularization and the dynamic system of bone tissue mineralization and demineralization. Other limitations include the standardization of protocols, model reproducibility, and the complexity of integrating different cell types into a single device.

Future prospects focus on two main directions: technological integration and clinical application. The use of advanced microfluidic systems will allow precise simulation of the dynamic conditions typical of bone tissue, such as blood flow and biomechanical forces, enhancing model fidelity. Simultaneously, the use of patient-derived cells will enable the development of personalized models. Finally, the integration of highly sensitive biosensors into Organ-on-chip systems will provide real-time monitoring of key cellular processes, further boosting the ability of these technologies to support research and treatment of bone diseases.

The use of different cell types in modern culture techniques described in this review, represents a significant advancement in the study of bone tissue, overcoming the limitations of traditional methods. These approaches allow for a deeper understanding of the cellular and molecular dynamics involved in bone regeneration and pathology. In three-dimensional models, spheroids primarily utilize MSCs and osteoblasts, which, in multicellular aggregates, better replicate cell–extracellular matrix interactions and improve cell survival compared to two-dimensional models. Bone organoids, on the other hand, use both MSCs and iPSCs, enabling the three-dimensional reconstruction of bone tissue morphology and functions, making them ideal for studying complex processes such as osteogenesis and bone homeostasis. In microfluidic systems, the integration of MSCs, osteoblasts, and sometimes endothelial cells allows for the dynamic simulation of bone microenvironments, recreating physiological conditions such as vascularization and mechanical stimulations. The combination of these isotypes in controlled on-chip environments enables the development of highly physiological models, ideal for studying bone regeneration and evaluating new therapies.

In conclusion, the integration of multiple cellular isotypes into advanced three-dimensional culture and microfluidic techniques represents a breakthrough in bone tissue research, offering more realistic and reliable models that enhance the understanding of biological processes and accelerate the development of innovative regenerative therapies.

## Data Availability

The data presented in the review can be obtained from the corresponding author.

## Abbreviations

ECM: Extracellular Matrix
BTE: Bone Tissue Engineering
MSCs: Mesenchymal Stem Cells
iPSCs: Induced Pluripotent Stem Cells
hMSCs: Human Mesenchymal Stem Cells
hADSCs: Human Adipose-Derived Stem Cells
hBMSCs: Human Bone Marrow-Derived Stem Cells
hESCs: Human Embryonic Stem Cells
AF-MSCs: Amniotic Fluid Mesenchymal Stem Cells
DPSCs: Dental Pulp Stem Cells
PD-MSCs: Placental-Derived Mesenchymal Stem Cells
Runx2: Runt-Related Transcription Factor 2
Sp7: Osterix (Specificity Protein 7)
ALP: Alkaline Phosphatase
OCN: Osteocalcin
SPARC: Secreted Protein, Acidic and Rich in Cysteine
OPN: Osteopontin
TGF-β: Transforming Growth Factor Beta
BMPs: Bone Morphogenetic Proteins
SAOS-eGFP: SAOS (Human Osteosarcoma) Enhanced Green Fluorescent Protein
PLA: Polylactic Acid
PGA: Polyglycolic Acid
PLGA: Poly-(DL–lacto–co-glycolic acid) Copolymer
PCL: Polycaprolactone
SV40: Simian Virus 40
EVs: Extracellular Vesicles
HA: Hydroxyapatite
TCP: Tricalcium Phosphate
ZTA: Zirconia Toughened Alumina
CPC: Calcium Phosphate Cement
hUVECs: Human Umbilical Vein Endothelial Cells
DRG: Dorsal Root Ganglion
GelMA: Gelatin Methacryloyl
GO: Graphene Oxide
VEGF: Vascular Endothelial Growth Factor
BMP-2: Bone Morphogenetic Protein-2
SAOS-2: Sarcoma Osteogenic-2 (Human Osteosarcoma Cell Line)
OHS-4: Osteosarcoma Cell Line 4
HOS-TE-85: Human Osteosarcoma TE-85 Cell Line
MG-63: Human Osteosarcoma Cell Line MG-63
CAL72: Human Osteosarcoma Cell Line CAL72
KPD-XM: Osteosarcoma Cell Line KPD-XM
TPXM: Osteosarcoma Cell Line TPXM
Oct4: Octamer Binding Transcription Factor 4
Sox2-Sex: Determining Region Y-Box 2
Klf4: Kruppel-Like Factor 4
c-Myc: Cellular Myelocytomatosis
FGF2: Fibroblast Growth Factor 2
IGF1: Insulin Growth Factor 1
TRAP: Tartrate-Resistant Acid Phosphatase
